# Dendritic Cells and Their Immunotherapeutic Potential for Treating Type 1 Diabetes

**DOI:** 10.3390/ijms23094885

**Published:** 2022-04-28

**Authors:** Farhan Ullah Khan, Puregmaa Khongorzul, Ahmed Aziz Raki, Ashwini Rajasekaran, Denis Gris, Abdelaziz Amrani

**Affiliations:** Department of Pediatrics, Immunology Division, Faculty of Medicine and Health Sciences, Centre de Recherche du CHUS, Université de Sherbrooke, 3001, 12th Avenue North, Sherbrooke, QC J1H 5N4, Canada; farhan.ullah.khan@usherbrooke.ca (F.U.K.); puregmaa.khongorzul@usherbrooke.ca (P.K.); ahmed.aziz.raki@usherbrooke.ca (A.A.R.); ashwini.rajasekaran@usherbrooke.ca (A.R.); denis.gris@usherbrooke.ca (D.G.)

**Keywords:** type 1 diabetes, dendritic cells, tolerance, immunity, immunotherapy

## Abstract

Type 1 diabetes (T1D) results from the destruction of pancreatic beta cells through a process that is primarily mediated by T cells. Emerging evidence suggests that dendritic cells (DCs) play a crucial role in initiating and developing this debilitating disease. DCs are professional antigen-presenting cells with the ability to integrate signals arising from tissue infection or injury that present processed antigens from these sites to naïve T cells in secondary lymphoid organs, thereby triggering naïve T cells to differentiate and modulate adaptive immune responses. Recent advancements in our knowledge of the various subsets of DCs and their cellular structures and methods of orchestration over time have resulted in a better understanding of how the T cell response is shaped. DCs employ various arsenal to maintain their tolerance, including the induction of effector T cell deletion or unresponsiveness and the generation and expansion of regulatory T cell populations. Therapies that suppress the immunogenic effects of dendritic cells by blocking T cell costimulatory pathways and proinflammatory cytokine production are currently being sought. Moreover, new strategies are being developed that can regulate DC differentiation and development and harness the tolerogenic capacity of these cells. Here, in this report, we focus on recent advances in the field of DC immunology and evaluate the prospects of DC-based therapeutic strategies to treat T1D.

## 1. Introduction

Type 1 diabetes (T1D) is an autoimmune disease that mainly affects children and young adults but can develop at any age. This disease arises from the selective destruction of insulin-producing pancreatic beta cells through a process that is mediated by an autoimmune response resulting from the breakdown of autoimmune tolerance. Approximately 5–10% of all diabetic patients have T1D, including more than 500,000 children worldwide, mostly in Europe and North America [1]. Moreover, epidemiological data have demonstrated that the T1D incidence has amplified significantly in recent years [2]. In 2019, Diabetes Research and Clinical Practice declared 128,900 newly diagnosed T1D cases globally in individuals under 20 years of age [3]. Both environmental elements and genetic susceptibility play crucial roles in advancing T1D. Polymorphisms in the HLA region of the major histocompatibility complex (MHC) broadly define the genetic risk of emerging T1D. The most prevalent loci are HLA-DQ8 and HLA-DQ2, which are found in 90% of type 1 diabetic patients [4]. These HLA molecules are associated with an enhanced presentation of various beta-cell-derived peptides by antigen-presenting cells (APCs). In addition to the HLA region, the insulin (INS), cytotoxic T lymphocyte-associated protein 4 (CTLA-4), IL-2 receptor (IL2RA), and protein tyrosine phosphatase N 22 (PTPN22) genes have the most significant influences on the etiopathogenesis of T1D [5,6]. A variety of immune cells participate in the pathogenesis of T1D, involving innate and adaptive immune systems and leading to the expansion of self-reactive, antigen-specific B and T lymphocytes. These immune cells can trigger islet inflammation to induce insulitis, which evolves into diabetes.

Dendritic cells (DCs) are the body’s sentinels par excellence, which act as “conductors” of the immune response by “coordinating” signals from different parts of the immune system. Dendritic cells are motile cells with a stellate morphology that express high levels of MHC molecules and the integrin CD11c and are characterized by their ability to migrate from nonlymphoid to lymphoid organs and their superior ability to activate T lymphocytes [7]. DCs can take up various antigens, including micro-organisms released by dead cells, extracellular fluid, and apoptotic cells, which can be processed and present on MHC class I and class II molecules to naïve T cells in the form of peptides. DCs can be found throughout the human body. They can form a diverse network to sense homeostatic discrepancies and process antigens for presentation to T cells, thereby establishing an interface between innate and adaptive immune systems. In addition, DCs can secrete growth factors and cytokines that modulate ongoing immune responses and are influenced by their connections with other immune cells, such as natural killer (NK) cells and innate lymphoid cells (ILCs) [8,9].

At present, DCs are believed to be a diverse cell population whose members vary in ontogeny, anatomical locality, relocation, cytokine production pattern, and immunological responses. They are situated in nonlymphoid tissues, where they screen the surrounding environment via their pattern recognition receptors (PRRs) and identify pathogen-associated molecular patterns (PAMPs) [10]. Once DCs capture antigens, they travel to lymphoid organs and then dispose of the antigens to T lymphocytes. Thus, DCs contribute to the regulation of immune responses through effector T cell lineages and immune tolerance by producing different patterns of cytokines [11,12].

## 2. Dendritic Cell Ontogeny

**Figure 1:**
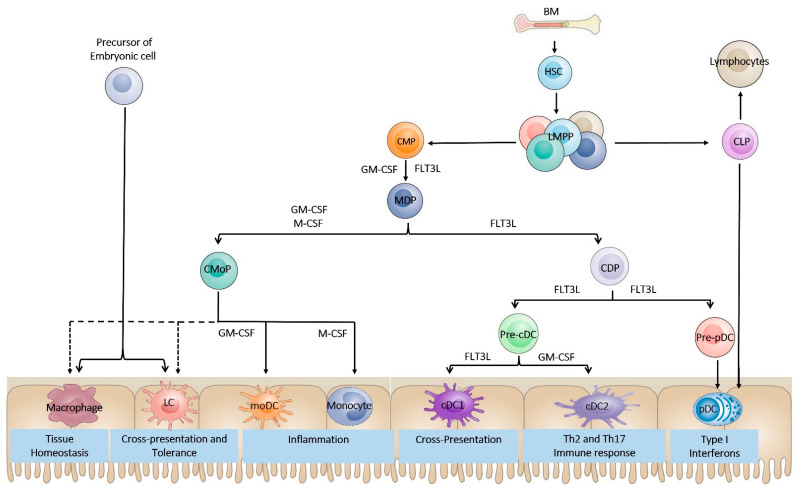
Ontogeny of functionally specialized dendritic cell subsets. Dendritic cells (DCs) originate from hematopoietic stem cells (HSCs) in the bone marrow (BM) that transit into lymphoid-primed multipotent progenitors (LMPPs). LMPPs differentiate into common myeloid progenitors (CMPs) and common lymphoid progenitors (CLPs). CMPs then branch into common DC progenitors (CDPs), which give rise to plasmacytoid DCs (pDCs), a major producer of type I interferons, and conventional DC (cDCs), whose primary function is to prime naïve T cells into common monocyte progenitors (CMoPs), which are committed to the monocyte, macrophage, and Langerhans cell (LC) lineages. CLPs give rise to pDCs and lymphocytes, such as T cells, B cells, and NK cells. A distinct macrophage lineage is derived from embryonic precursors and mainly generates tissue-resident macrophages and Langerhans cells, which can also be replaced over time by bone marrow monocyte-derived macrophages in different tissues, especially under inflammatory conditions. Tissue-resident macrophages maintain tissue homeostasis and are poor inducers of naïve T cells but are potent activators of B cells and are efficient at clearing apoptotic cells. Although LCs are categorized with macrophages on an ontogeny basis, they display many functional activities that overlap with cDCs. Monocyte-derived DCs (MoDCs), also known as inflammatory DCs (iDCs), are potent producers of TNF/iNOS (TIP) and are prominent at the site of inflammation. They typically execute functions in the tissues, such as antigen presentation to T effector cells, eradication of pathogens, and cytokine production. Stages at which key growth factors have been determined to be essential are indicated. MDP, macrophage and DC progenitor; Pre-pDC, pre-plasmacytoid DC; Pre-cDC, pre-conventional DC; cDC1, conventional type I DC; cDC2, conventional type II DC; GM-CSF, granulocyte-macrophage colony-stimulating factor; M-CSF, macrophage colony-stimulating factor; FLT3-L, Fms-like tyrosine kinase 3 ligand.

The classic model of DC development mainly comes from mouse research. DCs originate from bone marrow (BM) CD34^+^ hematopoietic stem cells (HSCs), which transit into the common myeloid progenitor (CMP) displaying a Lin^−^ c-Kit ^high^ Sca1^−^ IL-7R alpha^−^ subset and a common lymphoid progenitor (CLP) (Figure 1). The CMP differentiates into a bipotent progenitor called a macrophage and DC progenitor (MDP), giving rise to monocytes and DCs [13,14]. The MDP then begins to reduce the expression of c-Kit, an indication of differentiation into common dendritic cell progenitors (CDPs) displaying the Lin^−^c-Kit^int,^ Flt3^+^ M-CSFR^+^ phenotype. Similar to CDPs, a common monocyte progenitor cell (cMoP) was recently discovered downstream of the MDP that produces monocytes but not DCs [15]. CDPs can differentiate into precursors of classical or conventional DCs (Pre-cDCs) with the manifestation of the zinc finger and BTB domain containing 46 (ZBTB46) and ID2, while the expression of transcription factor 4 (TCF4) results in the generation of plasmacytoid DC precursors (Pre-pDCs) [16,17]. Pre-cDCs are recognized by the expression of Siglec-H, CD11c, SIRP alpha^low^, and MHC-II^int^, while Pre-pDCs can be identified by low expression of M-CSFR [18]. Pre-cDCs then further branch into cDC1 and cDC2 subsets, depending on the expression of key transcription factors related to each type (IRF8 and BATF3 for cDC1 or KLF4 and IRF4 for cDC2) [19,20,21]. In short, CDPs can differentiate into cDCs and pDCs (Figure 1). It is important to mention that Pre-cDC, Pre-pDC, CDP, MDP, and CMP cells are situated in the BM, while cDC1s, cDC2s, and pDCs are positioned in the periphery, such as in the lymphoid organs or blood [22].

During DC development and differentiation, growth factors, such as granulocyte-macrophage colony-stimulating factor (GM-CSF), Fms-like tyrosine kinase 3 ligand (Flt3-L), and macrophage colony-stimulating factor (M-CSF), are needed [23] (Figure 1). Flt3-L, which binds to the receptor Flt3, a protein tyrosine kinase receptor expressed especially in DC progenitors in the BM, is the most important growth factor involved in DC lineage diversification. Previously, it was shown that Flt3-L/Flt3 signaling is vital for developing and differentiating pDCs and cDCs in vitro [14,24,25]. Moreover, the in vivo role of Flt3 in DC development was demonstrated in Flt3-L deficient mice that showed severe insufficiency in DCs, and, to a lesser extent but also apparent, in mice deficient in their receptor CD135 (Flt3) or challenged with inhibitors of CD135 [26,27]. GM-CSF is another growth factor that is also involved in DC differentiation (Figure 1). GM-CSF is not necessary for steady-state DC differentiation, as demonstrated by a slight reduction in the number of DCs in mice lacking GM-CSF or GM-CSF receptors [28]. However, GM-CSF plays a crucial role in developing CD103^+^ CD11b^+^ DCs in the lamina propria, which is severely compromised in GM-CSF and GM-CSFR deficient mice [29]. In vitro, GM-CSF is a key factor for DC development from BM in mice and human monocytes while hindering the growth of pDCs in a STAT5-dependent manner [30]. On the other hand, M-CSF, which has a significant role in developing macrophages and monocytes, also participates in pDC differentiation from MCSFR^+^ precursor cells in the BM [31] (Figure 1). Nevertheless, despite the reduced levels of Langerhans cells (LCs) and monocytes in mice deficient in M-CSF and its receptor, no variations in the DC levels of lymphoid organs were noticed [32]. Moreover, M-CSF is necessary for the normal development of CD103^–^CD11b^+^ DCs in nonlymphoid tissues and can sustain the differentiation of pDCs and cDCs in the absence of FLT3 in cell culture [33].

## 3. Dendritic Cell Subsets

DCs were initially categorized into lymphoid and myeloid subsets, but this taxonomy does not precisely replicate each DC subgroup’s developmental origins (discussed previously [34]). Later, DC subgroups were classified based on their function, but DC plasticity, once again, defies rigid functional categories. In recent years, a new and simpler ontogeny classification scheme has emerged (reviewed previously in [35]), which is often associated with function (Figure 1). This categorizes DCs and related myeloid lineages into plasmacytoid DCs (pDCs), conventional (also identified as classical) DCs (cDCs), monocyte-derived DCs (MoDCs), and Langerhans cells (LCs). See Table 1 for details of the phenotypic markers that can be used to distinguish the different DC subtypes (Table 1). As the center of this report is DC-dependent type 1 diabetes treatment, here, we do not discuss the different DC subtypes, as the details are presented elsewhere [36,37,38].

## 4. Dendritic Cell Plasticity

**Figure 2:**
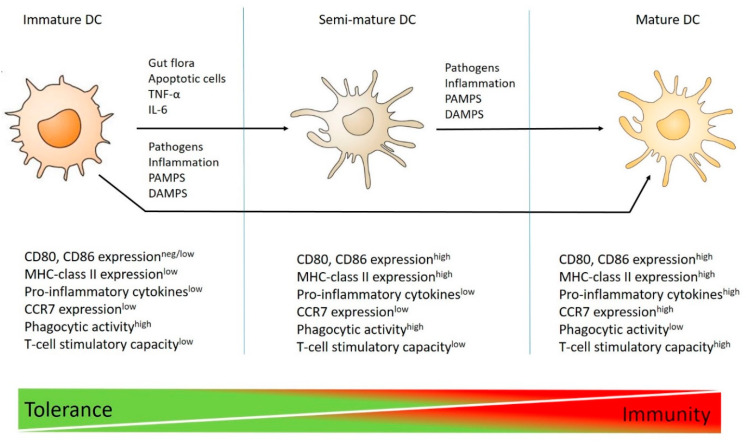
The classical scheme of different dendritic cell states in T cell tolerance and immunity. Dendritic cells (DCs) in the steady-state, i.e., in the absence of microbial or inflammatory signals, are immature and can internalize exogenous antigens and process them for MHC class-II-mediated presentation. However, they are devoid of strong upregulation of costimulatory molecules (CD80, CD86), MHC-class II, and proinflammatory cytokines; therefore, they cannot prime immune responses. Partial maturation results in elevated levels of costimulatory molecules and MHC-class II, but a lack or reduced level of proinflammatory cytokines gives rise to a DC population called semi-mature DCs. This population of DCs can be induced by lactobacilli from the gut flora, apoptotic cells, IL-6, or TNF-α. Both immature and semimature DCs prompt T-cell immune tolerance. Full DC maturation can be induced by extraneous factors, such as microbial or inflammatory signals, leading to downregulation of antigen acquisition and the antigen-processing ability, increased expression of CD80, CD86, and MHC-class II, and elevated levels of proinflammatory cytokines. All of these events result in T-cell priming and an increase in immunogenicity. TNF-α, tumor necrosis factor-alpha; IL-6, interleukin-6; MHC, major histocompatibility complex; PAMPS, pathogen-associated molecular patterns; DAMPS, damage-associated molecular patterns; CCR7, C-C chemokine receptor type 7.

Besides discovering DCs in 1973, Ralph Steinman and colleagues also highlighted their function in innate and adaptive immunity [39]. DCs are the most professional cell types that acquire and process antigens from pathogens and present them to the immune system [40,41]. The maturation status (immature, semimature, or fully mature) of DCs (Figure 2) governs the type of immune response generated to the presented antigen/peptide [42]. These three DC states have a series of independent special functions that enable them to exert different outcomes on the immune system.

Most DCs reside within the body in a so-called immature state (Figure 2). These immature DCs (iDCs) are usually regarded as tolerogenic DCs (tDCs). In this state, iDCs lack many features and processes that lead to a strong T cell response, such as increased MHC presentation, the expression of costimulatory molecules (CD80, and CD86), and the production of inflammatory cytokines, like IL-12, IL-23, and TNF-α, whereas they are efficient at detecting and sequestering antigens [43]. They can accumulate MHC class II molecules in the late endosome-lysosomal compartment and have their own set of chemokine receptors (for example, CCR7), anabling them to home to lympoide tissues [44,45]. Antigen recognition is carried out through different PRRs, such as Toll-like receptors (TLRs) and NOD-like receptors (NLRs), or indirectly through FcRs and complement receptors (CRs), which recognize antigen–antibody complexes and complements, respectively [46,47,48]. The main functional feature of iDCs is their endocytic and phagocytic ability, which occurs continuously under steady-state conditions [49]. As iDCs have reduced surface expression of costimulatory molecules, little chemokine receptor expression, and are deficient in releasing immunostimulatory cytokines, they induce immune tolerance through T effector cell anergy and the expansion of regulatory T cells. This immune tolerance is actively initiated and sustained by a combination of immune checkpoint pathways and the absence of costimulatory signals induced by DCs [50]. Immune checkpoint pathways are multiple inhibitory cascades that are essential for maintaining self-tolerance and regulating the duration/magnitude of the immune reaction. For example, DC-based ligands, such as CTLA-4 and programmed cell death protein ligand 1 (PD-L1), result in T cell unresponsiveness or immunosuppressive T cell differentiation [51].

Under steady-state conditions, most DCs in peripheral tissues display an immature phenotype in the absence of inflammatory or microbial signals. However, under certain conditions, such as in the presence of lactobacilli from the gut flora [52], intranasally applied ovalbumin (OVA) [53], apoptotic cells [54], or TNF-α [55], immature DCs can differentiate into an intermediate subset of DC maturation, called the semimature state (Figure 2). This subset of DCs has high expression of costimulatory molecules and MHC-class II; however, they are deficient in producing elevated levels of proinflammatory cytokines, such as TNF-α, IL-1β, IL-6, IL-12p40, and IL-12p70. In one study, it was shown that semimature DCs can be generated in an IL-6 dependent manner by treating bone-marrow-derived DCs (BMDCs) with the commensal bacterial strain Bacteroides vulgatus [56]. In line with this finding, similar outcomes have been observed with DNA-matured DCs in experimental collagen-induced arthritis [57], TNF-α-matured DCs in a murine model of thyroiditis [55], MyD88-silenced DCs, and LPS-matured DCs following intestinal allograft transplantation in a rat model [58].

In contrast to immature and semimature DCs, dangerous environmental signals (including inflammatory cytokines and microbial ligands) transform immature and semimature DCs into a fully mature state, where they are known as mature DCs (mDCs) (Figure 2). Maturation is related to a reduced antigen-capture ability, increased antigen processing and presentation via elevated expression of MHC class II, greater capacity to migrate to T-cell-rich areas, such as draining lymph nodes through the acquisition of the chemokine receptor CCR7, and increased ability to prime naïve T cells via enhanced cytokine production and costimulatory molecule expression [59]. The production of cytokines by mDCs is a vital component of the immune response, because these signaling molecules are indispensable to the differentiation of T cells. Moreover, ligation of the costimulatory receptor CD40 (also known as TNFRSF5) on DCs to CD40L on T cells is an important signal used to differentiate iDCs into full mDCs that can initiate adaptive T-cell-mediated immunity [60]. The interaction of antigen-specific T cells with mDCs leads to naïve T cell priming and subsequent differentiation into effector T cells with unique functions and cytokine profiles capable of initiating antigen-specific responses [61]. The crosstalk between mDCs and CD4^+^ T cells may result in the differentiation of CD4^+^ T cells into diverse T helper (Th) subpopulations, such as Th1 [62], Th2 [63], Th17 [64], or other CD4^+^ T cell subsets [65].

## 5. Metabolic Changes in DC during Development, Rest, and Activation

Growing evidence has emerged over recent years to support the notion that cellular metabolism is not only required to fulfill the energetic and biosynthetic demands that arise when immune cells switch from a quiescent to an activated state, but it also impacts or even dictates immune cell subset and function, activation, and differentiation, including DCs [66]. ATP, the main carrier of energy in cells, is generated by glycolysis and oxidative phosphorylation (OXPHOS), a process that involves metabolite intermediates. The latter are not only substrates for downstream biochemical reactions but can also act as signals that influence gene expression and, therefore, the outcome of the immune response. The generation of DCs from progenitor cells is linked to lipid metabolism and mitochondrial biogenesis, which are triggered by the peroxisome proliferator-activated receptor (PPAR) and PPAR co-activator 1 (PGC1) and aided by PPAR, mTOR, and MYC signaling [67,68]. Although pre-cDC1s and pre-cDC2s differentiate into immature cDC1s and cDC2s with unique transcriptional patterns, little was known about the metabolic distinction between cDC1s and cDC2s until recently. Studies of conventional DCs showed that the mitochondrial mass and membrane potential of cDC1s are greater than those of cDC2s [69] and that cDC1s display much greater oxidative phosphorylation (OXPHOS) than DC2s [70]. Overall, these data indicate that cDC1s and cDC2s have different metabolic profiles that are reflective of their distinct immune functions. Interestingly, during Flt3L-induced differentiation of bone-marrow-derived DCs (BMDCs), inhibition of AMP-activated kinase (AMPK) or fatty acid oxidation (FAO) was shown to promote cDC2 differentiation. However, DC differentiation was tilted toward the generation of cDC1s when reactive oxygen species (ROS) were inhibited [69]. Another research group found that depleting Tsc1 (a negative regulator of mTOR signaling) lowers the levels of cDCs and pDCs in vivo and leads to the differentiation of FLT3L-stimulated bone marrow cells into cDCs and pDCs, which is associated with dysregulated mitochondrial respiration, fatty acid synthesis, and glycolysis [71].

Differentiated DCs reside in peripheral tissues in a relatively quiescent or immature state (iDCs). Fatty acid oxidation (FAO) is the core metabolic pathway involved in iDCs. When triggered by immunosuppressive signals such as IL-10 [72] or IL-27 [73], tissue-resident iDCs can differentiate into tol-DCs. There is increasing evidence that metabolic programming underlies the tolerance of DCs. However, in tol-DCs, in contrast to the iDCs, cellular metabolism switches toward active oxidative phosphorylation (OXPHOS) with a reduction in glycolysis and maintenance of a high level of catabolic metabolism [74]. Previously, it was shown that tol-DCs’ regulatory activities are disrupted by FAO and OXPHOS inhibition and partially restore their immunostimulatory function [75]. On the other hand, activation of DCs (maturation) by Toll-like receptor (TLR) agonists, such as lipopolysaccharide (LPS), CpG or Poly(I:C), or by type I interferon (IFN), induces a metabolic switch from OXPHOS to glycolysis [76,77]. This results in an immediate increase in glycolytic flux via the associated pentose phosphate pathway, which is accompanied by increases in the spare respiratory capacity and fatty acid synthesis. Pharmacological blockade of glycolysis with 2-deoxyglucose (2-DG) results in the inhibition of DC maturation, as demonstrated by lower expression of costimulatory molecules (CD40 and CD86) and MHC-II as well as reduced DC survival [78]. Therefore, tolerogenic properties of DCs seem to rely less on glycolysis and more on OXPHOS. Moreover, this switch to glycolytic metabolism was found to be initiated by the activation of TANK-binding kinase 1 (TBK1), IκB kinase-ε (IKKε), and AKT, a pathway downstream of TLRs, which is crucial for DC migration and activation [79]. After being activated, DCs remain glycolytic by increasing the glycolysis components, including pyruvate kinase 2 (PKM2), lactate dehydrogenase (LDH), and phosphofructokinase (PFK). Other reports have shown that, in IL-10-induced tolerogenic DCs, the activation of AMPK by IL-10 impedes LPS-induced DC glycolysis and maturation, raising the notion that extrinsic paracrine signaling pathways might promote the formation of an immunotolerant milieu by altering DC metabolism [76]. Taken together, these studies imply that different metabolic profiles in DCs are crucial drivers of differential DC functions. Since therapy is a promising avenue to treat autoimmune diseases such as T1D, dissection of the immunometabolic mechanisms underlying DC immunogenic versus tolerogenic functions will open new tolerogenic DC-based therapeutic avenues for the treatment of autoimmune diseases like T1D.

## 6. Type 1 Diabetes and Dendritic Cells

Although beta cell dysfunction and beta-cell-targeted autoimmune processes are known to be involved in T1D, the precise etiology and pathological mechanisms are still largely yet to be elucidated. There are substantial data indicating that, in both humans and animal models of T1D, T cells are the major player involved in the development and progression of this disease. The loss of insulin-producing beta cells is mainly facilitated and orchestrated by CD8^+^ and CD4^+^ T cells specific to beta-cell antigens [80]. These T cells employ an array of processes to induce beta-cell destruction. CD8^+^ T cells can eliminate pancreatic beta cells via MHC class-I mediated cytotoxicity, while both CD8^+^ and CD4^+^ T cells secrete the inflammatory cytokine IFN-γ, thereby inducing the expression of the death receptor FAS (also called CD95) and the beta-cell production of chemokines [81]. Activation of FAS signaling through its binding to the FAS ligand expressed by activated diabetogenic CD4^+^ T cells can trigger beta-cell apoptosis [82]. Moreover, IFN-γ can induce macrophages to augment their secretion of proinflammatory cytokines, such as TNF-α and IL-1β. Compared with other endocrine cells in islets, beta cells express excessive levels of IL-1 receptors and tend to be more vulnerable to IL-1β-induced apoptosis through FAS induction. This crosstalk between macrophages and T cells undoubtedly aggravates immune-mediated beta cell stress and adds to their destruction.

In the early stages of diabetes, however, inflammation is characterized by an influx of DCs [83] into the islets in response to an anomaly that has yet to be identified, such as impaired islet architecture remodeling via apoptosis, cross-presentation of endogenous peptides in response to viral pathogens, or superantigen-driven immune responses [36,84]. Several studies have unveiled that DCs are responsible for inducing pathogenic beta-cell-specific T cells by presenting beta-cell antigens [85,86]. DCs and macrophages are the earliest detectable immune cells in the islets of the T1D animal model nonobese diabetic (NOD) mice at the age of 3 to 4 weeks [81,87]. Diana et al. demonstrated that IFN-γ producing plasmacytoid dendritic cells are recruited to the pancreas where they initiate diabetogenic T cell responses and the development of T1D in NOD mice [88]. Another study showed that NOD mice engineered to express TNF-α specifically in beta-cells using the rat insulin promoter exhibit increased DC accumulation in the islets which, in turn, present beta-cell antigens to CD4^+^ T cells, followed by massive destructive insulitis and the promotion of diabetes onset [89]. Several other studies also reported the pathological relevance of DCs in the induction and maintenance of T1D [90,91,92] (previously reviewed in [93]).

Numerous lines of evidence have revealed DC defects in the immunopathogenesis of T1D. It was shown that DCs derived from NOD mice and bio-breeding (BB) rats are dysfunctional in aspects ranging from the overactivation of DCs [85,94,95] to DC hypofunctionality [96,97]. Despite some recent studies indicating that possible deficiencies may also exist in humans [98,99], the response of diabetic patients to infection, recall to vaccine antigens, or capacity to induce hypersensitivity does not display any significant impairment [100,101], thereby creating more obscurity about potential defects in the development and function of DCs.

## 7. Dendritic Cell-Targeted Therapies for Treating T1D

In recent decades, our understanding of the immune system has made great strides and powerful therapeutic tools have been developed, notably targeted antigen delivery to DCs, fusion proteins, and monoclonal antibodies against countless receptors expressed on T cells and a series of cytokine milieus that have begun the era of targeted immune therapy. The fact that DCs play a pivotal role in inducing and maintaining self-tolerance makes them a desirable target for therapeutic intervention. Interestingly, a variety of immunosuppressive agents have been explored to treat T1D (reviewed previously in [102]). In this review report, we only highlight those interventions that have been examined for their effects on DC maturation and ability to treat T1D, which will likely broaden our knowledge in the field.

### 7.1. Costimulation Blockade

**Figure 3:**
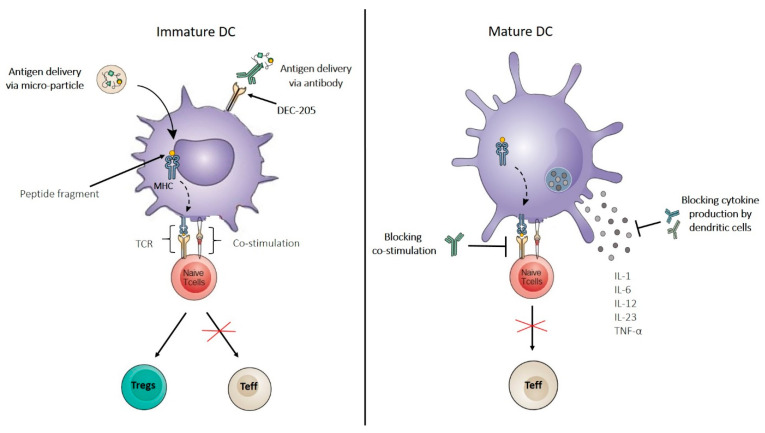
Dendritic-cell-targeted therapies for treating type 1 diabetes. Therapies targeting dendritic cells support the tolerogenic potential of dendritic cells. DCs can be made tolerogenic by targeted delivery of self-antigens by coupling them to antibodies raised against specific dendritic cell receptors, such as the DEC-205, or by targeted delivery via microparticles. Other potential therapeutic strategies aim to limit the immunogenicity of DCs by impeding their production of inflammatory cytokines or by reducing their expression levels of costimulatory molecules and, therefore, the induction of effector T cell responses. Most of these approaches implicate the usage of monoclonal antibodies, which target molecules that are selectively expressed by DCs. DEC-205, decalectin-205; MHC, major histocompatibility; TCR, T cell receptor; Tregs, regulatory T cells; Teff, T effector cells; IL, interleukin; TNF-α, tumor necrosis factor-alpha.

Costimulation is a crucial second signal that primes T cells after the first exposure to an antigen and is the link between adaptive and innate immunity. APCs, such as DCs, process and display antigen-derived peptides to the T-cell receptor (TCR) through the MHC peptide complex. However, in the absence of costimulation, T cells become unresponsive or may undergo apoptosis (Figure 3). DC surface receptor costimulatory molecules, for example, CD80/CD86 (also identified as B7-1 and B7-2), produce the necessary signals to initiate the induction and differentiation of naïve T cells and may inhibit immune tolerance, as occurs in T1D. CD80/CD86 can bind to CD28 on T cells (for autoregulation and intercellular association) as well as CTLA-4 produced by T cells (to attenuate immune suppression and cellular disassociation). The first drug targeting the binding of the CD80/CD86 costimulatory pair to CD28 was the fusion protein CTLA4-Ig, later identified as abatacept [103], a drug that has already been approved for treating rheumatoid arthritis patients [104,105].

Abatacept is a chimeric protein made up of the human CTLA-4 receptor conjugated with the modified Fc part of human IgG1 that is used as a decoy receptor for CD80/86 and prevents CD28-induced coactivation. The TrialNet research team studied the efficacy of abatacept in newly onset T1D patients (6–45 years old), in which the treatment group received 27 infusions of abatacept within a 2-year period [106] (Table 2). At the end of the treatment period, patients receiving abatacept showed significant C-peptide preservation compared with the placebo group (59% higher, *p* = 0.0029) after 24 months. However, after 6 months, preservation of the C-peptide declined, reaching the placebo level despite continuous treatment for 2 years. Investigation of peripheral T cell subpopulations by flow cytometry showed that naïve CD4^+^ T cells of abatacept-treated patients were moderately but significantly amplified, while the concentration of central memory CD4^+^ T cells decreased, and this seemed to be related to the preservation of the C peptide [107] (Table 2). Of some concern was a parallel and considerable reduction in the Treg cell percentage from baseline at 6, 12, and 24 months [107], which may have contributed to the finding that C-peptide responses started to decline soon after treatment began but at a slower rate than in the placebo group [106]. This decrease in Tregs can be attributed to the fact that Tregs, like other T cells, require costimulation to develop and exert their suppressive function [108]. Recently, it was demonstrated that this reduction in C-peptide preservation was associated with the transient elevation of activated B cells (that bind to abatacept) and reduced inhibition of anti-insulin antibodies [109] (Table 2).

Similarly, since CTLA-4 on the cell surface may be the main mechanism by which Tregs regulate APC function [110], there are still unresolved issues regarding the outcome of continuous therapy with soluble CTLA4-Ig on Treg functionality. Nonetheless, the abatacept trial conducted in T1D patients provided essential preliminary insights into the potential costimulatory blockade in T1D and is worthy of further investigation. A trial assessing the ability of abatacept in combination with rituximab (anti-CD20 monoclonal antibody) to prevent T1D in at-risk patients is currently ongoing (ClinicalTrials.gov identifier NCT03929601).

### 7.2. Blocking Cytokine Production

Cytokines produced by DCs activate and educate T cell differentiation and migration. When mature, DCs release a series of potent proinflammatory molecules, such as IL-12, IL-1, TNF-α, and IL-6 (Figure 3), which have been shown to have potent roles in T1D development [110]. Inhibiting the secretion of these molecules can induce noticeable changes in the preservation of pancreatic beta cell function [111].

The proinflammatory cytokines IL-1α and IL-1β, produced by DCs and/or macrophages, are potent immunomodulators that play key roles in pancreatic beta cell destruction [112,113]. IL-1 acts directly on beta cells, damaging the production and release of insulin and promoting cytokine- and hyperglycemia-induced beta-cell death [114]. In rodent models, it has been shown that IL-1 blockade results in slow progression and impairs the initiation of T1D [115]. IL-1 has been therapeutically targeted in a clinical trial in children newly diagnosed with T1D [116] (Table 2). In this clinical trial, fifteen children within 1 week of diagnosis of T1D received a daily IL-1 antagonist (Anakinra) for 28 days and were monitored for 6 months. The results demonstrated some significant outcomes in the protection against newly diagnosed T1D. Additional clinical trials in T1D patients also showed that IL-1 inhibition can induce pancreatic beta-cell preservation [117] (Table 2).

TNF-α is another well-known cytokine produced by DCs that acts as an intermediate molecule in autoimmune diseases. This cytokine is produced during the inflammation process and can trigger signaling cascades related to cell survival, the inflammatory response, apoptosis, and cell differentiation. The cytokine TNF-α binds to the receptor’s TNF-R1 and TNF-R2 to initiate its responses. The TNF-R1 receptor has a death domain, while TNF-R2 does not, but it can exacerbate the cytotoxic effect of TNF-R1. As a result of infection and inflammation, TNF-α is mainly released by immune cells, such as lymphocytes and DCs [118,119]. The binding of TNF-α with the TNF-R1 receptor can enhance NF-κB activation or activate the caspase pathway, which plays an essential role in the execution of programmed cell death or apoptosis [120]. NF-κB triggers the expression of genes that code for cytokines (e.g., INF-γ, IL-1, TNF-α, IL-6, IL-12, and IL-2) as well as the expression of molecules that regulate cell cycle progression, cell proliferation, and apoptosis, such as TNF-receptor-associated factor 1 (TRAF-1), TRAF-2, cellular inhibitor of apoptosis protein 1 (c-IAP1), c-IAP2, B-cell lymphocyte/leukemia-2 (Bcl-2), Fas, c-myc, and cyclin D1 [121,122]. Therefore, the blockage of TNF-α has been investigated as a therapeutic target in a clinical trial aimed at prolonging the endogenous release of insulin in pediatric patients newly diagnosed with T1D [123] (Table 2). This clinical trial was a randomized, double-blind, and placebo-controlled 24-week study in which eighteen patients (11 males and 7 females, aged 7.8–18.2 years) were randomly assigned to receive either etanercept (recombinant TNF-α receptor–IgG fusion protein) or a placebo. This pilot study demonstrated improved beta cell mass preservation (measured by the C-peptide levels) and reduced glycated hemoglobin levels.

One of the other important cytokines involved in autoimmune inflammation is IL-6. IL-6 can be released by a variety of cell types, including dendritic cells [124]. The pathological function of IL-6 in T1D is related to the IL-6R–gp130–STAT3 signaling axis. Signal transduction through this pathway is crucial for the differentiation of Th17 cells and inhibition of Treg cell development by inhibiting FOXP3 expression [125]. In a subset of T1D patients, IL-6 was found to be overexpressed [126], and as a result, anti-IL-6 therapy was initiated. The clinical trial EXTEND (clinical trial NCT02293837) investigated whether blocking IL-6 signaling (tocilizumab, anti-IL-6 receptor antibody) can provide improved beta-cell function in T1D patients. They found that, in newly diagnosed T1D patients, tocilizumab lowered T cell IL-6R signaling but unfortunately did not prevent the loss of residual beta cell function [127] (Table 2).

Another proinflammatory cytokine produced by antigen-presenting cells in response to PAMPs and DAMPs is IL-12. It is mainly released by DCs and phagocytes (monocytes/macrophages and neutrophils) in response to pathogens (viruses, bacteria, intracellular parasites, and yeast-like fungi) [128,129]. It induces immune response polarization toward the Th1 profile by inducing IFN-γ expression [130,131]. This cytokine is also considered a possible target for T1D therapy. In a clinical trial, T1D patients were tested for the application of Ustekinumab (IL12/23 blocking molecule) (ClinicalTrials.gov identifier NCT02117765) [132] (Table 2).

### 7.3. In Vivo Targeting of DCs

Generally, these types of therapy involve the use of agents that are deliberately targeted to a specific subset of DCs, but these methods can also include approaches that work by altering the local DC environment, although not directly targeting DCs (Figure 3). Treatment of NOD mice with nanoparticles (NPs) containing short antisense primary transcripts of the costimulatory molecules CD40, CD80, and CD86 has been shown to downregulate targeted receptors, induce a tolerogenic phenotype in DC populations, and prevent and/or reverse T1D [133]. The use of antigen-linked antibodies against the endocytic receptor DEC-205 (Figure 3) to deliver islet-specific antigens to DCs has been proven to be a promising strategy for treating T1D in NOD mice. Previously, DEC-205 was used to supply an IGRP206–214 mimotope to DCs to investigate its impact on highly diabetogenic T cells in NOD mice [134]. This study showed that IGRP206–214-loaded DCs significantly reduced the percentage and absolute number of diabetogenic IGRP-specific CD8^+^ T cells in pancreatic islets independently of the PD-1/PD-L1 pathway, resulting in the protection against T1D.

In a recent study, ~1 µm phagocytosable polylactic acid-glycolic acid ethanol (PLGA) microparticles (MPs) (Figure 3) were used to deliver tolerance-promoting factors such as vitamin D3, TGF-β1, GM-CSF, and T1D-specific autoantigen insulin to DCs to reprogram autoimmune responses and prevent autoimmunity [135]. This MP system successfully prevented 60% of prediabetic NOD mice from developing T1D by increasing the number of tDCs and the Treg cell population. Similarly, in another recent study, a targeted nanoparticle delivery system was used to deliver the antigen heat shock protein 65-6 × P277 (H6P) directly to the intestinal DCs of NOD mice through oral vaccination. This delivery system facilitated increased H6P uptake by DCs in gut Peyer’s patches and promoted the induction of the Th2 immune response and Treg upregulation, resulting in full protection from diabetes [136].

### 7.4. Ex Vivo Generation of Tolerogenic DC

**Figure 4:**
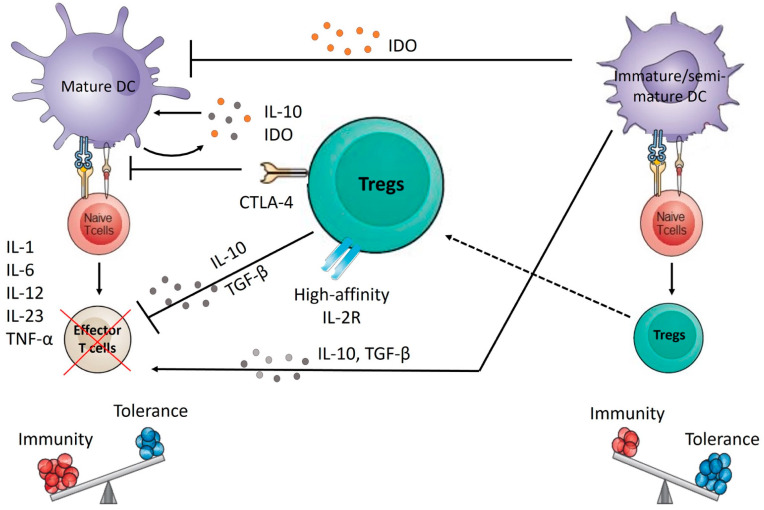
Schematic representation of dendritic cells and regulatory T cell interactions. Immature/semimature DCs secrete IDO impeding DC maturation as well as anti-inflammatory cytokines, such as IL-10 and TGF-β, inhibiting effector T cell activation. Immature/semimature DCs also give rise to Tregs, which contribute to immune tolerance by blocking the priming of effector T cells directly via IL-10 and TGF- β or indirectly through interactions with DCs and by blocking their maturation with the help of CTLA-4. CTLA-4 expressed on Treg has a higher affinity for CD80/86 molecules expressed on DCs than CD28 molecules expressed on effector T cells, meaning that Tregs competes with the effector T cells to bind CD80/CD86. DC CD80/CD86 and Treg-CTLA-4 interaction also results in the secretion of IL-10 and IDO, which contributes to the restraint of DC maturation. Treg can also preferentially sequester the T-cell proliferation factor IL-2 due to the high expression of constitutive IL-2R (CD25). The dotted line is used to emphasize that some differentiated Tregs use the above mechanisms to suppress effector T cells activation and balance immunity and tolerance. Tregs, regulatory T cells; IL-10, interleukin-10; TGF-β, tumor growth factor-beta; IDO, Indoleamine 2,3-dioxygenase.

Various methods aimed at controlling DC phenotypes have been explored to ensure that they retain a tolerogenic function and drive tolerance rather than immunity (reviewed previously [137]). These methods include challenging DCs with cytokines such as IL-10 [138], IL-10/TGF-β [139], TSLP [140], GM-CSF [141], pharmacological agents such as dexamethasone and vitamin D3 [142], carbon monoxide (CO) [143], anti-CTLA-4 antibody [144], and secretory IgA [145], among others. Generally, treating DCs with these agents results in an immature or semimature phenotype characterized by lowered expression of costimulatory molecules, and reduced production of inflammatory cytokines. These tolerogenic DCs secrete anti-inflammatory cytokines, like IL-10 and TGF-β, and a metabolite called IDO that inhibits effector T cell activation and DC maturation. They are also involved in the induction of the Treg differentiation (Figure 4). We previously reported that, in comparison with immunogenic BMDCs generated with GM-CSF and IL-4 (IL-4/DCs), BMDCs generated with GM-CSF (GM/DCs) acquire the signature of tolerogenic IL-10-producing DCs [146]. These GM/DC populations display an immature phenotype with a slight upregulation in CD80 but not CD86, CD40, or MHC-II expression and produce high levels of IL-10 and lower amounts of IL-12p70. GM/DCs also show a diminished ability to trigger diabetogenic CD8^+^ T cells to proliferate and effectively induce Treg conversion and expansion. Further research from our laboratory showed that, compared with immunogenic IL-4/DCs, the tolerogenic GM/DC subset alters the cytokine environment from Th1 toward Th2 cytokines and effectively prevents diabetes when injected into NOD mice [146,147,148]. In line with these results, we further confirmed, through in vitro studies, that NOD DCs genetically modified to express the active form of the Stat5b TF that mediates GM-CSF/GM-CSFR signaling acquire the signature of tolerogenic DCs [149,150]. These tolerogenic DCs were shown to be efficient at providing protection against T1D through an increase in the Treg pool and suppressive activity as well as through the promotion of Th2 and Tc2 immune responses.

Tolerogenic DCs have also been used in vitro to expand Treg cells, which can be adoptively transferred into patients to suppress or prevent inflammatory responses and autoimmunity. Tregs constitutively expresses the surface marker CTLA-4, which can interact with the DC costimulatory molecules CD80 and CD86 to block the CD28-dependent activation of effector T cells and activate the DC expression of IDO, TGF-β, and IL-10 (Figure 4), thereby further strengthening the tolerogenic phenotype of DCs [151].

Another approach using tolerogenic DCs for the prevention/treatment of T1D is to use antisense oligonucleotides to downregulate costimulatory molecule expression (CD40, CD80, and CD86) in DCs [152]. This approach has been found to substantially delay the onset of diabetes in NOD mice by increasing the concentration of Tregs [152]. Based on these encouraging data, a phase 1 clinical trial was initiated, which demonstrated the safety and tolerability of these tailored tolerogenic DCs in established T1D patients [153] (Table 2). A phase II follow-up clinical trial (ClinicalTrials.gov identifier NCT02354911) is currently underway that uses tolerogenic DCs isolated from patients with newly diagnosed T1D. A similar ongoing clinical study (ClinicalTrials.gov identifier NCT01947569) using tDCs with impaired costimulation has also been registered.

**Table 2:** Summary of the clinical trials mentioned in the text.

NCT Number	Recruitment Status	Study Date	Completion Date	Groups	Outcomes	Reference
NCT00505375	Completed	February 2008	May 2012	Interventional	At the end of the treatment, patients receiving abatacept showed significant C-peptide preservation compared with the placebo group (59% higher, *p* = 0.0029) at 24 months. However, after 6 months, C-peptide preservation declined to the placebo level, despite continuous treatment for 2 years.	[106,107,108,109]
NCT03929601	Suspended	February 2020	Ongoing	Interventional	Result not published.	
NCT00645840	Completed	March 2008	September 2009	Interventional	Anakinra-treated patients had similar glycated hemoglobin and MMTT responses but lower insulin requirements 1 and 4 months after diagnosis compared with controls and lower insulin-dose-adjusted glycated hemoglobin 1 month after diagnosis.	[116,117]
NCT00730392	Completed	October 2002	January 2008	Interventional	Treatment of pediatric patients newly diagnosed with type 1 diabetes with etanercept resulted in lower glycated hemoglobin and increased endogenous insulin production, suggesting the preservation of beta-cell function.	[123]
NCT02293837	Completed	March 2015	August 2020	Interventional	Tocilizumab reduced T cell IL-6R signaling but did not modulate CD4^+^ T cell phenotypes or slow the loss of residual β cell function in newly diagnosed individuals with type 1 diabetes.	[127]
NCT02117765	Unknown	March 2015	June 2017	Interventional	Ustekinumab was deemed safe to progress to efficacy studies at doses used to treat psoriasis in adults with T1D. A 90 mg maintenance dosing schedule reduced proinsulin-specific IFN-γ and IL-17A-producing T cells. Further studies are warranted to determine whether Ustekinumab can prevent C-peptide AUC decline and induce a clinical response.	[132]
NCT00445913	Completed	March 2007	February 2016	Interventional	Treatment with autologous dendritic cells in a native state or directed ex vivo toward a tolerogenic immunosuppressive state is safe and well-tolerated. Dendritic cells upregulated the frequency of a potentially beneficial B220+ CD11c2 B-cell population, at least in type 1 diabetes autoimmunity.	[153]
NCT02354911	Unknown	October 2015	January 2019	Interventional	Result not published	
NCT01947569	Unknown	October 2013	November 2013	Interventional	Result not published	
NCT04590872	Recruiting	April 2022	Ongoing	Interventional	Result not published	

Previously, it was shown that naturally derived proinsulin peptide C19-A3 is safe and capable of eliciting the immunoregulatory responses, such as the stimulation of IL-10 production and the increase of Tregs Foxp3 expression in type 1 diabetic patients [154]. Further studies showed that tolDCs presenting this peptide can induce proinsulin-specific regulatory T cells [155]. Based on these exciting outcomes, a phase 1 clinical trial was conducted in the Netherlands to assess the clinical safety and feasibility of proinsulin peptide-loaded tolDCs in nine patients with longstanding type 1 diabetes [156]. After tolDC therapy, all patients maintained tight glycemic control with constant HbA 1c levels and unaltered insulin needs. Most importantly, there was no induction of allergic reaction to insulin, no signs of systemic immune suppression, and no interference with insulin therapy, suggesting that this immune intervention therapy is feasible and safe. A complementary phase 1 follow-up clinical trial will also be conducted in the United States to investigate its therapeutic potential and side effects in T1D patients who use insulin and have no other diabetes-related health complications (Clinicaltrials.gov identifier: NCT04590872).

## 8. Future Views and Concluding Remarks

Given the exclusive nature of DCs found at the interface between innate and adaptive immune responses, they provide target cells for clinical intervention in T1D patients. Diverse DC subtypes use different transcription factors [157] so that these DC subtypes can be embattled differently for immunomodulation. One can think of the possibility of combining tDCs and Tregs as coimmunization or the serial administration of cellular immunotherapy in autoimmunity, especially in newly onset T1D. The use of autologous tDCs in conjunction with patient Tregs can stabilize Foxp3 expression and its genomic locus. Because tDCs are often seen to release IL-10, TGF-β, and retinoic acid [158,159], stable Tregs will, in turn, affect the tDC tolerance status through intercellular interactions and paracrine immunomodulatory cytokines, which may result in much more effective and long-term protection from diabetes.

Moreover, in cancer research, the area of DC-based vaccines is much more advanced, and several clinical studies have already been carried out. Many characteristics of cancer DC vaccines have been investigated, and it is obvious that features, such as the conditioning regime of DCs, the antigen form used to pulse DCs, and the means of administration all play vital roles in defining the end result of DC-based therapy. These features need to be considered as more DC-based therapies for T1D treatment are proposed. Several clinical trials have been conducted to induce antigen-specific tolerance in T1D patients [160]. These trials employ islet-specific antigens, for example, GAD65, insulin, or hsp70, and have tested multiple administration routes, such as oral, intranasal, and intradermal administration. To date, despite the evidence of immune tolerance observed in some cases, these trials have not had a significant impact on the disease [160]. It is predicted that the success of these trials will depend on APCs, most likely DCs, targeted by these antigen formulations. At present, in these trials, less attention has been given to the nature of antigen-presenting DCs; therefore, a deeper understanding of how DCs affect the development of T1D will aid in the advancement of novel therapeutic approaches.

## Figures and Tables

**Table 1:** Phenotypic markers of dendritic cell subsets.

DC Subset	DC Type	Human	Mouse	Transcriptional	TLR	Antigen	Major
		Markers	Markers	Factors		Presentation	Cytokines
pDC	Lymphoid-	CD123^+^	CD11b^−^	TCF4	1, 2, 4	Poor	Type I IFN
	resident DC	CD303^+^	CD11c^+^	IRF8	6, 7, 8, 9		
		CD304^+^	CD45RA^+^	E2.2			
		ILT3^+^	SIGLEC-H^+^				
		ILT7^+^	CD8α^+^				
		DR6	CCR7^+^				
cDC1	Lymphoid-	CD141^+^	CD11b^−^	BATF3	1, 2, 3, 4	Cross presentation	L-12p70
	resident DC	Clec9a^+^	CD11c^+^	IRF8	6, 8, 9, 10	on MHC-class I	IFN-λ
		CADM1^+^	CD103^+^	ID2			
		CXR1^+^	CD45RA^−^	NFIL3			
		BTLA^+^	CD8α^+^				
		CD11b^−^	CXR1^+^				
cDC2	Migratory DC	CD11b^+^	CD11b^+^	IRF4	2, 4, 5	Presentation on	?
		CD11c^+^	CD11c^+^	PU.1	6, 7, 8, 9	MHC-class II	
		CD1c^+^	CD45RA^−^	Notch2			
		SIRPα^+^	SIRPα^+^				
		Clec4a^+^	CD4^+^				
		Clec10a^+^	CD8α^−^				
		CX3CR1^+^	CX3CR1^+^				
Monocyte-	Induced by	CD11c^+^	CD11b^+^	KLF4	1, 2, 3	Cross presentation	TNF/iNOS
derived DC	inflammation	CD1a^+^	CD11c^+^	IRF8	4, 5,7, 8		
		CD1c^+^	LY6C^+^	PU.1			
		SIRPα^+^	CD8α^−^				
		CD206^+^	CCR2^+^				
Langerhans Migratory DC		CD1a^+^	CD11b^+^	ID2	1, 2, 3	Presentation of	IL-10
cells		CD207^+^	CD45RA^−^	RUNX3	5, 6, 10	self-antigens for	
		CD123^+^	CD8α^−^	β-catenin		tolerance induction	
		TROP2^+^	CXCL10^+^				

DC, dendritic cell; TLR, toll-like receptor; pDC, plasmacytoid DC; ILT3, Immunoglobulin-like transcript 3; DR6, Death receptor 6; SIGLEC-H, sialic acid-binding immunoglobulin-like lectin H; CCR7, C-C motif chemokine receptor 7; TCF4, transcription factor 4, IRF8, interferon regulatory factor 8; IFN, interferon; cDC1, conventional DC1; clec9a, C-type lectin-like receptor member (Clec) 9a; CADM1, Cell adhesion molecule 1 gene; CXR1, CX- chemokine receptor 1; BTLA, B- and T-lymphocyte attenuator; BATF3, basic leucine zipper transcription factor ATF-like 3; ID2, DNA binding protein inhibitor 2; NFIL3, nuclear factor interleukin 3 regulatory protein; MHC, major histocompatibility complex; IL-12, interleukin 12, cDC2, conventional DC2; SIRPα, signal regulatory protein alpha; CX3CR1, CX3C- chemokine receptor 1; Notch2, neurogenic locus notch homolog protein 2; KLF4, kruppel-like factor 4; TNF/iNOS, tumor necrosis factor/induced nitric oxide synthase; TROP2, Trophoblast cell surface antigen 2; CXCL10, C-X-C motif chemokine ligand 10; ID2, Inhibitor of DNA binding 2; RUNX3, RUNX family transcription factor 3.

## Data Availability

Not applicable.
